# High freezing sensitivity of legumes relative to other herbaceous species in northern temperate plant communities

**DOI:** 10.1093/aob/mcae072

**Published:** 2024-05-14

**Authors:** Samuel L Rycroft, Hugh A L Henry

**Affiliations:** Department of Biology, University of Western Ontario, 1151 Richmond St. N, London, ON N6A 5B7, Canada; Department of Biology, University of Western Ontario, 1151 Richmond St. N, London, ON N6A 5B7, Canada

**Keywords:** Fabaceae, frost, nitrogen fixers, snow removal, winter

## Abstract

**Background and Aims:**

Reduced snow cover and increased air temperature variability are predicted to expose overwintering herbaceous plants to more severe freezing in some northern temperate regions. Legumes are a key functional group that may exhibit lower freezing tolerance than other species in these regions, but this trend has been observed only for non-native legumes. Our aim was to confirm if this trend is restricted to non-native legumes or whether native legumes in these regions also exhibit low freezing tolerance.

**Methods:**

First, we transplanted legumes (five non-native species and four native species) into either an old field (non-native) or a prairie (native) and used snow removal to expose the plots to increased soil freezing. Second, we grew plants in mesocosms (old field) and pots (prairie species) and exposed them in controlled environment chambers to a range of freezing treatments (control, 0, −5 or −10 °C) in winter or spring. We assessed freezing responses by comparing differences in biomass, cover and nodulation between freezing (or snow removal) treatments and controls.

**Key Results:**

Among legume species, lower freezing tolerance was positively correlated with a lower proportion of nodulated plants and active nodules, and under controlled conditions, freezing-induced reductions in above-ground biomass were lower on average in native legumes than in non-native legumes. Nevertheless, both non-native and native legumes (except *Desmodium canadense*) exhibited greater reductions in biomass in response to increased freezing than their non-leguminous neighbours, both in controlled environments and in the field.

**Conclusions:**

These results demonstrate that both native and non-native legumes exhibit low freezing tolerance relative to other herbaceous species in northern temperate plant communities. By reducing legume biomass and nodulation, increased soil freezing could reduce nitrogen inputs into these systems.

## INTRODUCTION

In northern temperate regions, freezing stress limits the persistence and productivity of many plant species (Yadav, 2010). Even if plants survive freezing, damage to overwintering or perennating structures (Ambroise *et al*., 2020; Lubbe *et al*., 2021) can lead to a broad range of sub-lethal effects (Steponkus *et al*., 1993; Malyshev and Henry, 2012*a*, *b*). With climate change, increases in winter temperature variability (Orsenigo *et al*., 2014; Vincent *et al*., 2018) and changes in winter precipitation (Mekis and Vincent, 2011; Ahmed *et al*., 2022) are changing soil freezing dynamics over winter. Despite the occurrence of warmer, shorter winters, exposure to more intense soil freezing and increases in soil freeze–thaw cycles are occurring in some northern temperate regions where snow cover, which can insulate soil, is becoming more intermittent (Groffman *et al*., 2001; Christensen *et al*., 2007; Henry, 2008). These increases in soil freezing severity (i.e. lower soil temperatures, deeper soil frost and an increased frequency of freeze–thaw cycles) could have particularly important implications for the condition of overwintering herbaceous plants, which typically die back above ground and have their overwintering organs (e.g. shoot bases and root systems) positioned at or below the soil surface (Rapacz *et al*., 2014). The timing of freezing exposure also can have an important influence on the severity of freezing damage (Joseph and Henry, 2008; Bokhorst *et al*., 2009, 2011). With increased variability in winter temperatures, plants may de-acclimate prematurely, leaving them vulnerable to subsequent cold periods (Vyse *et al*., 2019); for example, false spring events, when above-seasonal temperatures are abruptly followed by a return to low temperatures in late winter (Peterson and Abatzoglou, 2014), can damage plants substantially (Marino *et al*, 2011; Allstadt *et al*., 2015).

Accounting for interspecific variation in freezing tolerance can be vital for predicting future shifts in plant community composition in northern temperate regions in the context of a changing climate (Kreyling, 2010; Kreyling *et al*., 2012; Komac *et al*., 2015; Smith and Sheridan, 2018). Changes to freezing regimes can influence ecosystem functioning considerably if specific plant functional groups are impacted disproportionately. Legumes (family: Fabaceae) often play a significant role in improving soil fertility (Vitousek and Howarth, 1991; Craine *et al.*, 2002; Palmborg *et al*., 2005; Barker *et al*., 2022) because most legumes form mutualisms with diazotrophic (i.e. nitrogen-fixing) rhizobia (de Faria *et al*., 1989; Sprent *et al*., 2017). In studies of freezing tolerance in northern temperate plant communities, a trend has emerged of legumes being more susceptible to freezing than other herbaceous groups (i.e. graminoids and non-leguminous forbs). For example, in a northern temperate old field, *Trifolium pratense* cover decreased more than non-leguminous forb cover in response to increased soil freezing initiated by snow removal (Lubbe and Henry, 2020). Similarly, *Securigera varia* biomass was reduced substantially more than that of both graminoids and non-leguminous forbs within plant–soil mesocosms exposed to increased spring freezing (Joseph and Henry, 2008). Rycroft (2023), who conducted snow removal experiments in both native and non-native plant communities, observed that low freezing tolerance of legumes in these systems was only present in non-native species, which may be poorly adapted to cold winters (Long *et al.*, 2021). However, the evidence for higher freezing tolerance in native legumes was based on only a single native species. It therefore remains necessary for the freezing tolerances of non-native legumes relative to native legumes in northern temperate herbaceous communities to be explored more systematically.

Although the mechanisms that explain the low freezing tolerance of non-native legumes remain uncertain, it has been demonstrated that freezing can have a negative effect on nodulation and nodule activity (Prévost and Bromfield, 1991; Prévost *et al.*, 1999), which can reduce the quantity of nitrogen (N) fixation (Lorin *et al.*, 2016; Thurston *et al.*, 2022) and subsequently reduce the competitive ability of legumes. Freezing tolerance can vary greatly among rhizobial strains or species (Drouin *et al.*, 2000; Prévost *et al.*, 2003), and the pairing of legumes with adequately freeze-tolerant rhizobia may therefore be a crucial component of legume freezing tolerance. Such a mechanism would imply that the presence of suitable freeze-tolerant rhizobial partners may be lacking for many non-native legumes, and that their presence must be assured for the successful establishment of legumes (native or non-native) in planting and restoration efforts in these regions.

The main objective of our study was to confirm whether the trend of low legume freezing tolerance in northern temperate plant communities is restricted to non-native legumes or whether native legumes in these regions also exhibit low freezing tolerance. First, we conducted snow removal experiments, with five non-native legume species transplanted into an old field plant community dominated by non-native species, and four native legume species transplanted into a tallgrass prairie dominated by native species, to assess the freezing tolerances of non-native and native legumes in the context of their respective herbaceous plant communities. We hypothesized that non-native legumes would exhibit significant decreases in biomass production under snow removal, whereas their neighbouring non-leguminous plants, along with the native legumes and their non-leguminous neighbours, would not. Second, we conducted freezing trials in controlled environment chambers with the same nine target legume species to test responses to freezing in winter versus late spring and to examine freezing effects on nodulation and nodule activity. We anticipated the most pronounced plant biomass effects would occur in response to spring freezing, and we also hypothesized that freezing would reduce the presence and activity of root nodules, but only in non-native legumes.

## METHODS

### Snow removal experiments with legumes transplanted into pre-existing plant communities

Snow removal experiments were conducted within two plant communities at the Environmental Sciences Western (ESW) field station near Ilderton, Ontario, Canada (43°04ʹ29.2″N, 81°20ʹ16.0″W). The first plant community, an old field, featured a mix of native and non-native herbaceous species, whereas the second community, a restored tallgrass prairie, comprised predominantly native herbaceous species. The soils of both communities were characterized as a Bryanston silt loam (Hagerty and Kingston, 1992). The dominant species in the old field were goldenrod (*Solidago* spp.), common dandelion (*Taraxacum officinale* Weber) and red clover (*Trifolium pratense* L.); species such as thistles (*Cirsium* spp. and *Sonchus* spp.), American asters (*Symphyotrichum* spp.), wild carrot (*Daucus carota* L.) and bird’s-foot trefoil (*Lotus corniculatus* L.) also were abundant in patches within the site. The dominant species in the tallgrass prairie plots were goldenrod (*Solidago* spp.) and wild bergamot (*Monarda fistulosa* L.), with native C4 grasses (e.g. *Andropogon gerardii* Vitman), American asters (*Symphyotrichum* spp.), rosinweeds (*Silphium* spp.), sedges (*Carex* spp.), showy ticktrefoil [*Desmodium canadense* (L.) DC.] and coneflowers [*Rudbeckia* spp. and *Ratibida pinnata* (Vent.) Barnhart] also abundant.

**Fig. 1.**
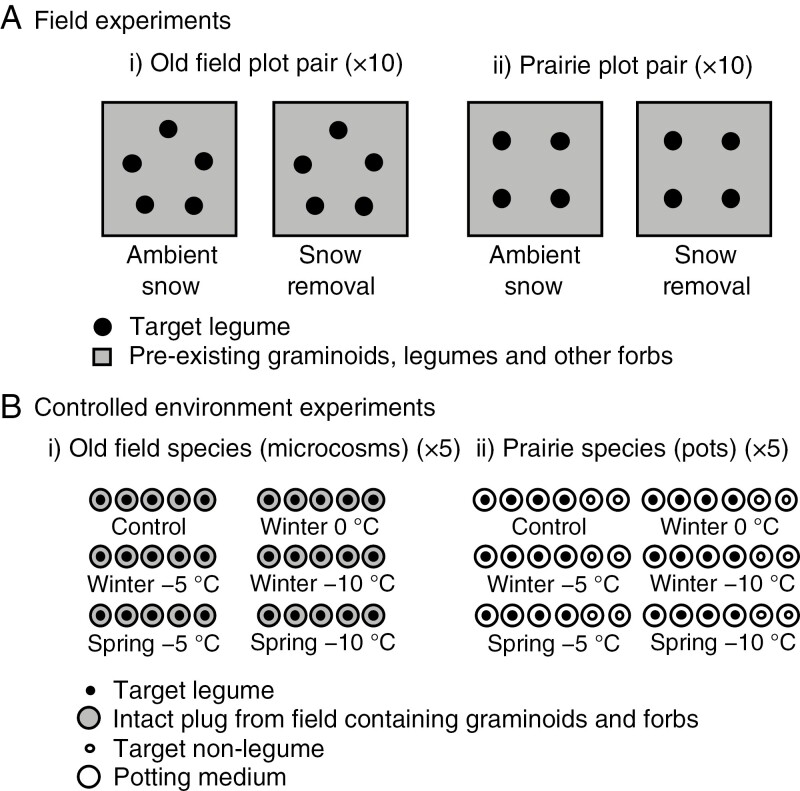
Experimental units and treatments used for (A) the field experiments (i, old field plots; ii, prairie plots) (*n* = 10) and (B) the controlled environment experiments (i, microcosms containing old field species; ii, pots containing prairie species (*n* = 5)).

In addition to assessing the snow removal responses of the pre-established plant communities, target legume individuals also were transplanted into the study plots to allow a greater diversity of legumes to be examined while simultaneously controlling for both age and size uniformity of the target individuals (Fig. 1A). To prepare the transplants, nine species (five non-native and four native) of perennial herbaceous legumes were grown in a glasshouse at the University of Western Ontario. The non-native species were *Lotus corniculatus* L. (bird’s-foot trefoil), *Melilotus officinalis* (L.) Pall. (yellow sweet clover), *Securigera varia* (L.) Lassen, *T. pratense* and *Trifolium repens* L. (white clover). Seeds of *L. corniculatus*, *M. officinalis*, *T. pratense* and *T. repens* were purchased from DLF Pickseed Canada, Inc. (Lindsay, Ontario, Canada), and seeds of *Securigera varia* were purchased from Ernst Seeds (Meadville, PA, USA). The establishment of these non-native species in the study region has often been the result of prior escape from cultivation or purposeful planting, but they have become naturalized in the area (Turkington *et al*., 1978; Turkington and Franko, 1980; Turkington and Burdon, 1983; Symstad, 2004; Ogle *et al*., 2008). The native species were *D. canadense*, *Desmodium paniculatum* (L.) DC, *Lespedeza capitata* Michx. and *Lespedeza hirta* (L.) Hornem. Seeds of the native legumes were obtained from Mary Gartshore, a local naturalist. Each species was broadcast sown into shallow trays containing Pro-Mix BX Mycorrhizae soil medium (Premier Horticulture Inc., Quakertown, PA, USA). Three weeks after germination, 60 individuals of each species were transplanted into 9-cm-wide, 9-cm-long and 13-cm-deep pots. Plants were watered daily *ad libitum* then transplanted (27 May – 3 June 2019) into plots among the pre-existing vegetation in the respective herbaceous plant communities (i.e. non-native legumes were transplanted into the old field to compare with the responses of other non-native herbaceous species, and native legumes were transplanted into the prairie to compare with the responses of other herbaceous native species). The five transplants in the old field plots were equally spaced in a pentagonal pattern and the four transplants in the prairie plots were equally spaced in a square pattern.

The snow removal experiments were conducted using a randomized block design (adapted from Lubbe and Henry, 2020), with 20 1 × 1-m plots designated within each plant community. Two plots were included in each block, with one plot randomly assigned to the snow removal treatment and the other assigned to the control (i.e. ambient snow cover) treatment. Those assigned to the snow removal treatment were subjected to increased soil freezing for the winter of 2019–2020, with snow removed via shovelling. All plots were covered with winter wrap (a white plastic netting) in mid-December to delineate the appropriate snow removal depth and prevent damage or removal of surface litter during snow removal. Snow removal was conducted when adequate snow accumulation (≥5 cm) coincided with sufficiently low air temperatures (≤−10 °C). Snow removal was conducted seven times from 20 January 2020 to 28 February 2020. Soil temperature loggers (LogTag TRIX-8, MicroDAQ, NH, USA) were placed 2 cm beneath the soil surface of snow removal and control plots in each site (three loggers per treatment per site) to measure soil temperatures hourly. The plastic netting was removed from all plots on 3 April 2020.

Compared to the 1991–2020 climate normal, the 2019–2020 winter (December to the end of February) was warmer on average (−1.6 °C vs. −4.1 °C) with normal total precipitation (218 mm vs. 221 mm), and there were 80 d with snow on the ground (Environment Canada., 2022; London Airport, 7 km from the study site). Although average soil temperature in the old field at 2 cm depth from December to the end of March was similar among the snow removal treatment (1.9 °C) and control (2.1 °C), snow removal resulted in a lower minimum soil temperature (−2.2 °C vs. −0.4 °C) and an increased number of freeze–thaw cycles (11 vs. 6). Similarly, in the prairie, average soil temperature at 2 cm depth was similar among the snow removal treatment (1.7 °C) and control (1.9 °C); however, snow removal resulted in a lower minimum soil temperature (−2.2 °C vs. −0.6 °C) and an increased number of freeze–thaw cycles (11 vs. 6).

Throughout the following growing season, percentage above-ground cover for each species in each plot was assessed through visual surveys (9–14 June 2020, 16–19 July 2020 and 16–19 August 2020). Above-ground biomass was harvested from each plot at peak plant growth (17 August 2020 for the old field, and 9–10 September 2020 for the prairie). First, the transplants were harvested individually. Then, an electric hedge trimmer was used to collect a 60 × 40-cm subsample of the remaining vegetation from each plot. The latter was then separated by functional group (non-leguminous forbs, NLF; leguminous forbs, LF; graminoids, GR); LF comprised naturally occurring legumes and was analysed separately from the transplanted legumes. For the prairie, some naturally occurring legumes were non-native, but most were the native species *D. canadense*. The harvested plant matter was dried at 60 °C for at least 72 h prior to weighing.

### Controlled environment experiments

Controlled environment freezing experiments were conducted on the target legume species, and non-leguminous plants for comparison, to explore their responses to variation in the timing and intensity of freezing. For the non-native species, each target legume was transplanted into an intact plant–soil microcosm, containing pre-established plants, collected from the field (Fig. 1B, i) (method adapted from Joseph and Henry, 2008). However, for the native species, the large size of the prairie plants precluded the collection of plant–soil mesocosms. Therefore, to examine freezing effects on native species, individual plants (target legumes and non-legumes) were grown separately in pots (Fig. 1B, ii).

For the non-native species, a total of 175 plant–soil microcosms were extracted from an old field at the Elginfield Observatory (43°11ʹ33″N, 81°18ʹ57″W) using 10-cm-diameter by 10-cm-high sections of PVC pipe. The microcosms contained a mix of pre-established species; the dominant graminoids were *Bromus inermis* Leyss, *Festuca rubra* L., *Poa* spp. and *Carex blanda* Dewey, the dominant forbs were *Conyza canadensis* L., *Solidago* spp., *Daucus carota* L. and *Erigeron annuus* L., and the sole abundant leguminous forb was *Securigera varia*. All plant–soil microcosms were transported to the University of Western Ontario (UWO) glasshouses. To prepare the transplants, seeds from each legume species were sown into shallow trays containing Pro-Mix BX Mycorrhizae soil medium (Premier Horticulture). Fifty individuals per species were transferred to 9-cm-wide, 9-cm long and 13-cm-deep pots 3 weeks after germination. All plants were watered daily *ad libitum* prior to transplantation. Each microcosm was then assigned randomly to one of the five non-native legumes species, with a single individual of the assigned non-native legume species transplanted into the centre of the microcosm (35 mesocosms per target legume species).

The native legumes were germinated using the same procedure as the non-native species, then the target plants were transferred to 15-cm-wide, 15-cm-long and 17-cm-deep pots to mitigate these plants becoming root-bound. In addition, individuals of two non-leguminous species, *Solidago canadensis* (a non-leguminous forb present in many herbaceous communities of the region) and *Andropogon gerardii* (a C4 graminoid present in restored and remnant tallgrass prairies in the region), were collected from the study site in autumn and transplanted into the same sized pots for comparison with the freezing responses of the native legumes.

The microcosms/pots for each target species of legume were randomly assigned to either a winter or spring freezing regime or a control treatment (*n* = 5). In November 2019, following senescence of above-ground plant matter, all plant–soil microcosms and pots were placed outside within cold frames adjacent to the UWO glasshouses and buried with soil up to the lip of the mesocosm or pot, then loosely covered with an ~2-cm-deep layer of wheat straw to mimic plant cover and protect the pots from excessive freezing during the non-treatment periods. Four data loggers were placed 2 cm beneath the soil surface and also covered with straw.

For the control (i.e. ambient) treatment, plants remained outdoors for the duration of the experiment, where they were exposed to the normal, ambient snow cover. For the winter and spring freezing regimes, plants were randomly assigned to either 0, −5 or −10 °C (winter; 3–6 March 2020) or −5 or −10 °C (spring; 16–19 May 2020) freezing treatments, with individual plants only experiencing a single winter or spring freezing treatment. All freezing treatments were conducted for 72 h in darkness in controlled environment chambers and temperatures below freezing were adjusted at a rate of 1 °C h^−1^. Following freezing treatment, the microcosms and pots were returned to the cold frames and re-buried and covered with straw. Straw was removed from the cold frames on 25 May 2020 to avoid further impacts on above-ground growth, and the plants remained in the cold frames until harvest.

Biomass for non-native legumes (and neighbouring non-legumes within the respective plant–soil microcosms) was harvested on 4–20 July 2020 (depending on the target legume species), with plant material from the microcosms separated out to the species level where possible (and subsequently pooled to the functional group level). Plant material was then separated into above- and below-ground biomass. Biomass harvesting for native legumes from the pots (and for comparative non-legume species) was conducted on 13–18 August 2020. Biomass was dried for at least 72 h at 60 °C. For both native and non-native legumes, the number and activity of symbiotic nodules was recorded. Nodule activity was assessed for a subset of five nodules for each surviving plant; if the nodule interior (i.e. bacteroid tissue) was red or pink it was deemed active, while nodules that were white, grey or green were deemed inactive.

### Statistical analyses

We assessed snow removal effects within each community on total above-ground biomass, functional group biomass and the above-ground biomass of individual legume species (for the pre-established plants), and the above-ground biomass of individual transplanted legume species, using two-way analysis of variance (ANOVA; species or functional group and snow removal as fixed factors and plot pair added as a random factor). These analyses were followed by paired one-tailed *t*-tests to assess reductions caused by snow removal within each species or functional group. For the pre-established plants within each community, snow removal effects on total cover and functional group cover, and the cover of the dominant species (i.e. those in all or nearly all plots) were assessed using repeated-measures ANOVAs (species or functional group, snow removal and month as fixed factors, and plot pair and plot ID added as random factors), followed by paired one-tailed *t*-tests to assess reductions caused by snow removal within each species or functional group for each date. For the controlled environment experiments, biomass (above- and below-ground biomass) was analysed for each species or functional group using either a one-way ANOVA and Games–Howell tests (if mortality among treatment groups resulted in substantially unequal sample sizes) or a one-way ANOVA and Tukey’s post-hoc analysis when survival was uniform. In addition, differences in freezing responses between native and non-native legume species were examined by calculating the mean above-ground biomass response ratio (treatment divided by control) for each species, then comparing the species means for natives versus non-natives using *t*-tests for each combination of freezing temperature and season. For plant nodulation, species means for natives versus non-natives also were compared using *t*-tests for each combination of freezing temperature and season. Correlations between mean above-ground biomass response ratio and the mean number of nodulated plants for each species also were examined for each combination of freezing temperature and season. All analyses were conducted using JMP version 17 (SAS Institute).

## RESULTS

### Transplant experiment – non-native legumes

**Fig. 2.**
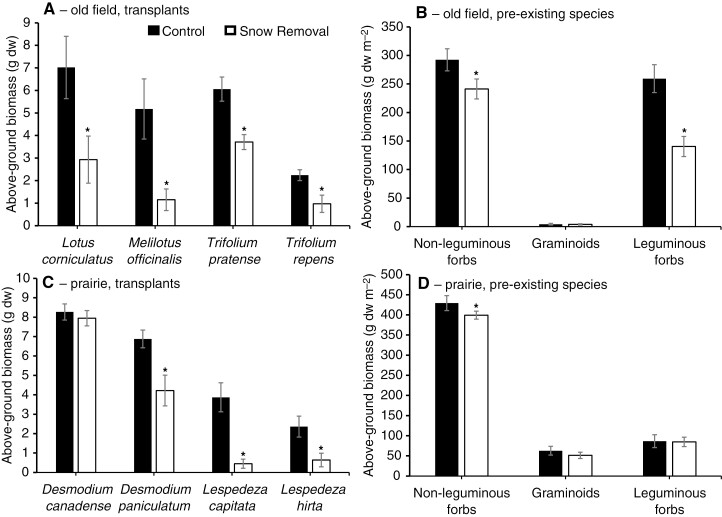
Mean above-ground biomass (± s.e.) of target herbaceous legumes and functional groups of pre-existing plants from ambient snow (dark bars) and snow removal plots (light bars) within the old field site and prairie site (*n* = 10 per treatment group). (A) Transplanted legumes and (B) functional groups (excluding transplants) within the old field site; (C) transplanted legumes and (D) functional groups (excluding transplants) within the prairie site. Asterisks indicate a significant difference (*P* < 0.05; paired one-tailed *t*-test) between treatments for each species/functional group.

Within the old field, the biomass of transplanted legume species decreased significantly by 39–78% in response to snow removal (ANOVA *P* < 0.001; paired *t*-tests *L. corniculatus*: *P* = 0.04; *M. officinalis*: *P* = 0.004; *T. pratense*: *P* = 0.0003; *T. repens*: *P* = 0.01; Fig. 2A; *S. varia* was not included in the biomass analyses because it experienced high winter mortality in both the control and treatment plots). At the whole-plot functional group level (not including transplants), there was a significant interaction between snow removal and functional group (ANOVA *P* < 0.001), explained by there being no snow removal effect on graminoid biomass (GR: *P* = 0.44) but significant snow removal effects on the other two functional groups, and a larger decrease in biomass in the leguminous forbs than in the non-leguminous forbs (LF: ~46% reduction, *P* < 0.001; NLF: ~18% reduction, *P* = 0.005; Fig. 2B). The whole-plot legume biomass response was driven primarily by established (i.e. non-transplanted) *T. pratense*, which exhibited 51% less biomass in the snow removal plots than in the control plots (*P* < 0.0001). The cover results were consistent overall with the biomass results (i.e. a significant interaction between snow removal and functional group; ANOVA *P* < 0.001), except the non-leguminous forbs increased significantly in cover in June and August in response to snow removal (Table 1).

**Table 1.** Mean proportional cover (± s.e.; a proportion of 1 = 100%) responses of herbaceous functional groups under both control (i.e. ambient snow) and snow removal (SR) treatments (*n* = 10 per treatment) within the old field and prairie sites across three visual surveys throughout the 2020 growing season.

		June		July		August	
Site	Functional group	Control	SR	Control	SR	Control	SR
Old field	GR	0.03 ± 0.01	0.03 ± 0.02	0.03 ± 0.01	0.03 ± 0.02	0.03 ± 0.01	0.03 ± 0.01
	LF	0.32 ± 0.01	0.23 ± 0.03*	0.31 ± 0.02	0.26 ± 0.03	0.33 ± 0.02	0.22 ± 0.02***
	NLF	0.66 ± 0.01	0.76 ± 0.02**	0.68 ± 0.02	0.73 ± 0.03	0.64 ± 0.02	0.75 ± 0.02***
Prairie	GR	0.08 ± 0.01	0.13 ± 0.03*	0.07 ± 0.01	0.12 ± 0.03	0.09 ± 0.01	0.11 ± 0.02
	LF	0.23 ± 0.02	0.12 ± 0.01***	0.23 ± 0.01	0.12 ± 0.02***	0.17 ± 0.02	0.13 ± 0.01*
	NLF	0.67 ± 0.02	0.74 ± 0.03*	0.69 ± 0.02	0.76 ± 0.03*	0.73 ± 0.02	0.75 ± 0.02

Functional group codes: GR = graminoids, LF = leguminous forbs, NLF = non-leguminous forbs. ANOVA revealed a significant interaction between treatment and functional group (*P* < 0.001). Asterisks indicate a significant difference between treatments within functional groups and survey period (*P < 0.05, **P < 0.01, ***P < 0.001; paired one-tailed *t*-test).

### Transplant experiment—native legumes

For the transplanted legumes in the prairie, there was a significant interaction between species and snow removal for above-ground biomass (ANOVA *P* = 0.018); the biomass of transplanted legume species decreased significantly in response to snow removal by 38–88%, with the exception of *D. canadense* (*D. canadense*: *P* = 0.19; *D. paniculatum*: *P* = 0.02; *L. capitata*: *P* = 0.002; *L. hirta*: *P* = 0.008; Fig. 2C). At the whole-plot functional group level (not including transplants), non-leguminous forbs decreased significantly by 8% in response to snow removal (NLF: *P* = 0.04), but neither graminoids nor leguminous forbs responded significantly (GR: *P* = 0.12; LF: *P* = 0.42; Fig. 2D). Nevertheless, the plot-level legume response was driven mostly by pre-established (i.e. non-transplanted) individuals of *D. canadense*, for which biomass did not differ between the control and snow removal plots (*P* = 0.47). For plant cover, there was a significant interaction between snow removal and functional group; ANOVA *P* < 0.001). However, contrary to the biomass results, the plot-level cover of non-leguminous forbs increased significantly in response to snow removal, whereas leguminous cover decreased significantly in response to snow removal (Table 1). There also was a significant increase in graminoid cover in the prairie in June (Table 1).

### Controlled environment experiment – non-native legumes

**Fig. 3.**
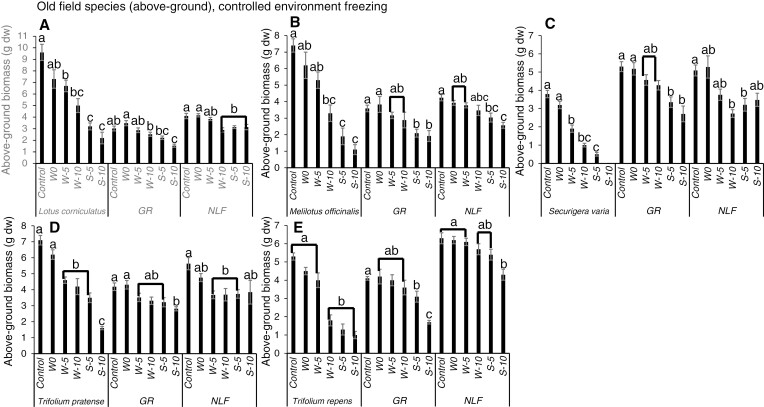
Mean above-ground biomass (g dw ± s.e.) of non-native legumes and non-leguminous neighbours in mesocosms subjected to different freezing severity treatments in a controlled environment over winter (W) and spring (S). Different letters indicate significant differences among treatments within a given species or functional group (Tukey’s HSD, *P* < 0.05, for groups with equal sample sizes; Games–Howell tests, *P* < 0.05, for groups with unequal sample sizes). NLF = non-leguminous forb, GR = graminoid.

**Fig. 4.**
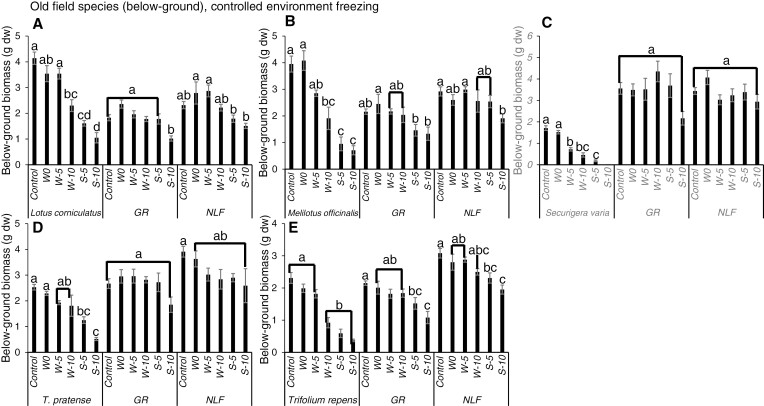
Mean below-ground biomass (g dw ± s.e.) of non-native legumes and non-leguminous neighbours in mesocosms subjected to different freezing severity treatments in a controlled environment over winter (W) and spring (S). Different letters indicate significant differences among treatments within a given species or functional group (Tukey’s HSD, *P* < 0.05, for groups with equal sample sizes; Games–Howell tests, *P* < 0.05, for groups with unequal sample sizes). NLF = non-leguminous forb, GR = graminoid.

In response to the winter freezing treatments, all of the non-native legumes exhibited significant decreases in above-ground biomass, and in almost all cases they were more sensitive to freezing than their neighbouring graminoids and non-leguminous forbs (Fig. 3). While *L. corniculatus* decreased significantly in response to −5 and −10 °C over winter, neither the graminoid nor non-leguminous forb neighbours of this target legume responded significantly to a winter freezing treatment (Fig. 3A). Similarly, the above-ground biomass of *S. varia* decreased significantly in response to −5 and −10 °C over winter, while its graminoid neighbours did not respond significantly to winter freezing and its non-leguminous forb neighbours only decreased significantly in response to −10 °C over winter (Fig. 3B). Likewise, the above-ground biomass of *T. pratense* decreased significantly in response to −5 and −10 °C over winter, while its graminoid neighbours did not respond significantly to winter freezing and its non-leguminous forb neighbours decreased significantly in response to −5 and −10 °C over winter (Fig. 3D). *Melilotus officinalis* and *T. repens* only decreased significantly in response to −10 °C over winter, but neither the graminoid nor non-leguminous forb neighbours of these target legumes responded significantly to a winter freezing treatment (Fig. 3C, E). The significant below-ground biomass responses of *S. varia* and *T. repens* were the same as for above-ground biomass, whereas unlike their above-ground responses, *L. corniculatus* and *M. officinalis* below-ground biomass only responded significantly to the most severe winter freezing treatment (−10 °C), and *T. pratense* below-ground biomass did not respond significantly to any winter freezing treatment (Fig. 4). Similarly, for all target legumes, the below-ground biomass of the neighbouring graminoids or non-leguminous forbs did not respond significantly to winter freezing (Fig. 4).

For the spring freezing treatments, the above-ground biomass of all target non-native legume species decreased significantly in response to both −5 and −10 °C, and these effects were more severe than the responses to the same temperatures over winter (Fig. 3). In most cases, the graminoid and non-leguminous forb neighbours also decreased significantly in response to the spring freezing treatments. However, the magnitudes of these decreases were larger in the legumes than in the graminoids and non-leguminous forbs (Fig. 3). Similar responses were observed for below-ground biomass as were observed for above-ground biomass for both the leguminous and non-leguminous species (Fig. 4).

### Controlled environment experiment – native legumes

**Fig. 5.**
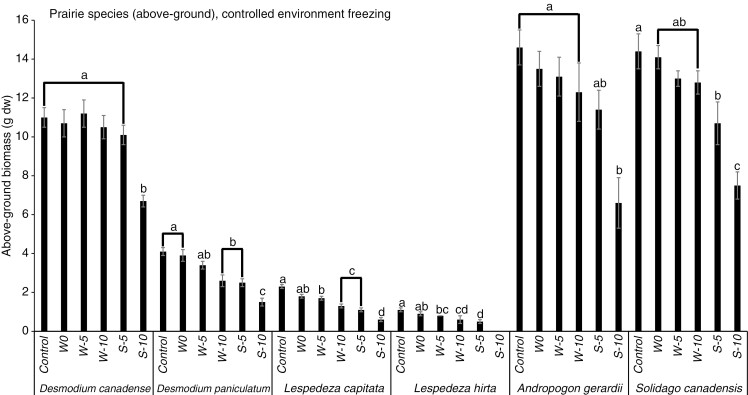
Mean above-ground biomass (g dw ± s.e.) of native species subjected to different freezing treatments in a controlled environment over winter (W) and spring (S). Different letters indicate significant differences among treatments within a given species or functional group (Tukey’s HSD, *P* < 0.05, for groups with equal sample sizes; Games–Howell tests, *P* < 0.05, for groups with unequal sample sizes).

The above-ground biomass of the native legume *D. canadense* did not respond significantly to the winter freezing treatments; however, two other native legumes, *L*. *capitata* and *L. hirta*, both decreased significantly in response to −5 and −10 °C, and *D. paniculatum* decreased significantly in response to −10 °C (Fig. 5). In contrast, the above-ground biomass of neither the prairie grass (*A. gerardii*) nor the non-leguminous forb (*S. canadensis*) responded significantly to the winter freezing treatments (Fig. 5). The effects of spring freezing on above-ground biomass were more severe than those of winter freezing for all species (Fig. 5). However, while *A. gerardii* and *D. canadense* only responded significantly to spring freezing at −10 °C, the other four species responded significantly to both −5 and −10 °C (Fig. 5).

**Fig. 6.**
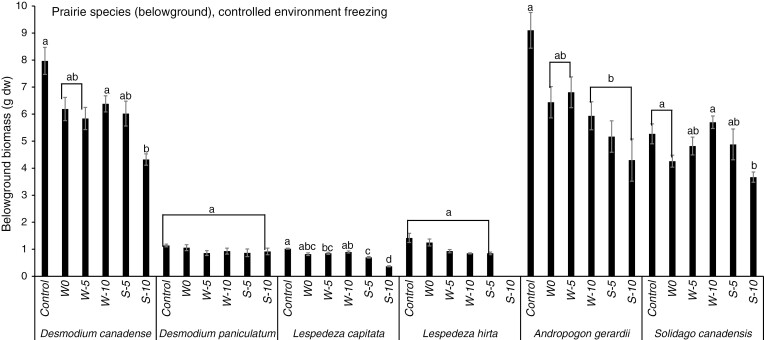
Mean below-ground biomass (g dw ± s.e.) of native species subjected to different freezing treatments in a controlled environment over winter (W) and spring (S). Different letters indicate significant differences among treatments within a given species or functional group (Tukey’s HSD, *P* < 0.05, for groups with equal sample sizes; Games–Howell tests, *P* < 0.05, for groups with unequal sample sizes).

Similar to the non-native species, the below-ground biomass of the native species was less sensitive than the above-ground biomass to freezing (Fig. 6). For winter freezing, the only significant below-ground biomass responses occurred for the legume *L. capitata* (−5 °C) and the graminoid *A. gerardii* (−10 °C) (Fig. 6). For spring freezing, there were significant reductions in below-ground biomass for the native legumes *D. canadense* (−10 °C), *L. capitata* (−5 and −10 °C) and *L. hirta* (for the latter, all died at −10 °C). The below-ground biomass of the native grass *A. gerardii* decreased significantly in response to spring freezing at both −5 and −10 °C, and the non-leguminous forb *S. canadensis* responded significantly to spring freezing at −10 °C (Fig. 6).

### Controlled environment experiments – nodulation

**Fig. 7.**
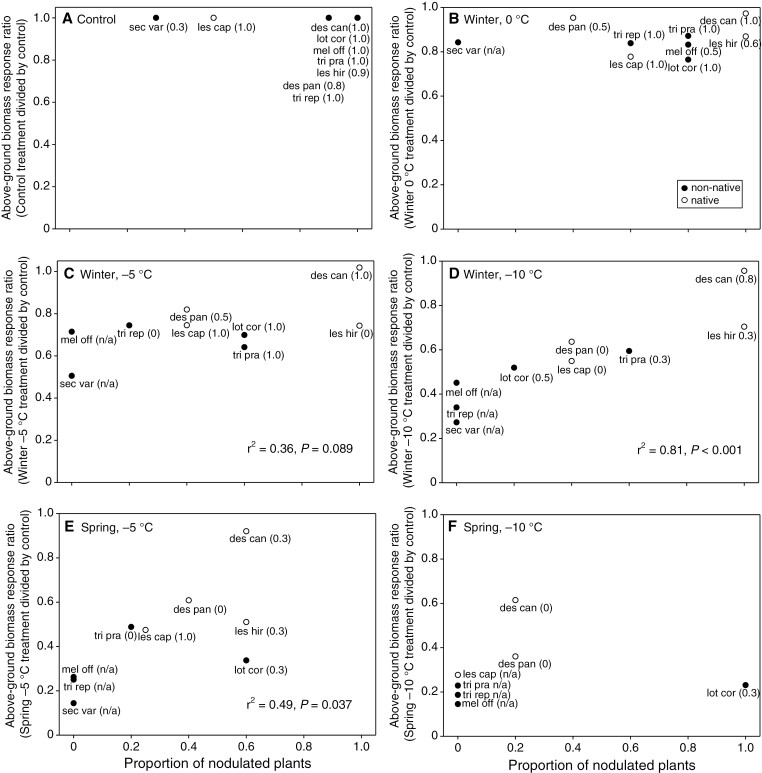
Above-ground biomass response ratios (treatment divided by control) as a function of the number of nodulated plants in response to (A) control, (B) winter freezing at 0 °C, (C) winter freezing at −5 °C, (D) winter freezing at −10 °C, (E) spring freezing at −5 °C and (F) spring freezing at −10 °C. Species codes are derived from the first three letters of the genus and species names combined, and the proportion of nodulated plants with active nodules are shown in parentheses. Closed circles denote non-native species and open circles denote native species.

Both the proportion of nodulated plants and the proportion of nodules that were active decreased with decreasing freezing temperature, with the most severe effects for a given freezing temperature occurring in the spring (Fig. 7). Among species, decreases in above-ground biomass relative to the control treatment were correlated with a decreased proportion of nodulated plants for winter freezing at −10 °C (*r*^2^ = 0.81, *P* < 0.001; Fig. 7D), spring freezing at −5 °C (*r*^2^ = 0.49, *P* = 0.037; Fig. 7E) and marginally significant for winter freezing at −5 °C (*r*^2^ = 0.36, *P* = 0.089; Fig. 7C). In addition, compared to native species, non-native species exhibited both larger decreases in above-ground biomass relative to the control treatment and a lower proportion of nodulated plants for winter freezing at −5 °C (*P* = 0.038 and *P* = 0.052; Fig. 7C), winter freezing at −10 °C (*P* = 0.022 and *P* = 0.023; Fig. 7D) and spring freezing at −5 °C (*P* = 0.018 and *P* = 0.038; Fig. 7E).

## DISCUSSION

We hypothesized that non-native legumes would exhibit significant decreases in biomass production in response to snow removal, whereas their neighbouring non-leguminous plants, along with the native legumes and their non-leguminous neighbours, would not. This hypothesis was supported to some extent at the whole-plot level by the responses of the pre-existing species, in that the non-native legumes, but not the native legumes, decreased substantially in response to snow removal. However, the pre-existing legumes in the native prairie plant community were dominated by a single species, *D. canadense*. The transplant component of the experiment revealed that the *D. canadense* response to snow removal was an anomaly, and that all the other native legume species examined were highly sensitive to freezing. Overall, the results of the snow removal experiment therefore support the observation that herbaceous legumes exhibit higher freezing sensitivity than other herbaceous species in northern temperate regions (Joseph and Henry, 2008; Lubbe and Henry, 2020). Moreover, contrary to our hypothesis, they reveal that such responses are not driven solely by non-native legume species.

The responses of legumes in the controlled environment experiments were generally consistent with those of the transplant experiments, with both non-native and native legumes (except *D. canadense*) exhibiting relatively low freezing tolerance. Nevertheless, reductions in above-ground biomass in response to freezing were larger on average in non-native than in native species, with the exception of the 0 °C treatment in winter, which caused little freezing damage, and −10 °C in spring, which damaged most species extensively. Moreover, the correlation among species between freezing-induced reductions in above-ground biomass, nodulation and nodulation activity provided insight into the possible mechanism underlying our observation of low legume freezing tolerance. Specifically, while reductions in nodulation and nodule activity have been documented previously as a response to freezing (Prévost *et al*., 2003; Liu *et al*., 2019), the presence of suitable rhizobial partners upon exposure to a stressor also may play an important role in improving plant stress responses, both in general (Villanueva, 2021; Liu *et al.*, 2022) and in response to freezing (Irshad *et al.*, 2021; D’Amours *et al.*, 2022). In our experiments, the number of nodulated plants and nodule activity were high for most species for the control treatment, which indicates poor legume freezing tolerance did not result from an absence of rhizobia prior to freezing. Nevertheless, as described above, freezing tolerance can vary greatly among rhizobial strains or species (Drouin *et al.*, 2000; Prévost *et al.*, 2003), and it has been demonstrated that reduced plant stress tolerance can arise from a lack of optimal rhizobial partners, even when (sub-optimal) rhizobial partners may be present (Grman *et al*., 2020). This mechanism might explain the high freezing tolerance of *D. canadense* in our snow removal experiments, given it was the only transplanted prairie species that was already abundant in the field, and thus may have had access to optimal rhizobial partners. Likewise, the presence of optimal rhizobial partners might explain why the native legumes in general exhibited lower reductions in biomass and nodulation than the non-native legumes in response to freezing. However, we only assessed nodule activity and did not characterize which rhizobial partners were present. Our study also did not account for other possible mechanisms of reduced freezing tolerance in legumes, including cyanogenesis (Olsen and Ungerer, 2008), susceptibility to soil frost heave (Lubbe *et al.*, 2021) and the disruption of tripartite symbioses (i.e. legume–rhizobia–mycorrhizal associations; Larimer *et al.*, 2014; Ossler *et al.*, 2015).

The controlled environment experiments revealed further important facets of legume freezing responses. Foremost, freezing-induced reductions in biomass were most pronounced for a given freezing temperature treatment in spring (as observed previously in Meyer and Badaruddin, 2001; Joseph and Henry, 2008), and legumes were generally more sensitive to freezing than other species at this time. High sensitivity to freezing in spring can result from plants experiencing cold de-acclimation prior to the cessation of frost events (Vankoughnett *et al.*, 2016). Premature cold de-acclimation in early spring is therefore a mechanism that may explain the high freezing sensitivity of legumes in northern temperate regions. Nevertheless, given that most of the legumes we examined also exhibited low freezing tolerance relative to the non-leguminous species over winter, at a time when all species would have been fully cold acclimated, the relatively low freezing tolerance of legumes could not be explained by premature cold acclimation in spring alone.

The controlled environment experiments also revealed that for several of the legume species (e.g. *D. paniculatum* and *T. pratense*) reductions in biomass in response to freezing primarily occurred above ground, with little or no effect on below-ground biomass. Allocation to below-ground biomass is particularly important given the role of below-ground structures in ensuring persistence of herbaceous plants over winter (Bertrand and Castonguay, 2003; Rapacz *et al*., 2014; Vyse *et al*., 2019; Lubbe *et al*., 2021). Plants may preferentially allocate resources below ground to ensure future winter survival at the expense of reduced above-ground productivity over the current growing season. Nevertheless, in some cases, a reduced reproductive effort following freezing damage to reproductive structures can allow for increased resource allocation to vegetative shoot growth (Tolvanen, 1997; Malyshev and Henry, 2012*b*). Plant root systems also may be exposed to less severe freezing than shoot bases because many roots are positioned deeper in the soil, where there is less penetration of frost. However, such depth-driven effects would not have occurred in our experiment, because the soil in the pots and soil mesocosms was frozen uniformly with soil depth.

## CONCLUSIONS

Our results demonstrated that the trend of high freezing sensitivities of herbaceous legumes relative to non-legumes in northern temperate plant communities not only applies to non-native legumes, but it also applies to many native legume species. Given the key functional role of legumes in N fixation, reduced legume biomass in response to altered soil freezing dynamics could reduce N inputs in these ecosystems in the context of a changing climate. Our finding that successful nodulation was associated with freezing tolerance in these species suggests further exploration is needed to establish whether the optimization of legume inoculation with compatible freeze-tolerant rhizobial species could improve the freezing tolerance and persistence of legumes in plant community management and restoration applications.
